# Correlation of Bioimpedance Spectroscopy with Risk Factors for the Development of Breast Cancer-Related Lymphedema

**DOI:** 10.1089/lrb.2017.0078

**Published:** 2018-12-13

**Authors:** Frank Vicini, Chirag Shah, Pat Whitworth, Michael Walker, Jing Shi

**Affiliations:** ^1^ Michigan Healthcare Professionals, 21st Century Oncology, Farmington Hills, Michigan.; ^2^ Department of Radiation Oncology, Cleveland Clinic, Taussig Cancer Institute, Cleveland, Ohio.; ^3^ Nashville Breast Center, Nashville, Tennessee.; ^4^ Walker Bioscience, Carlsbad, California.

**Keywords:** bioimpedance, breast cancer, lymphedema

## Abstract

***Background:*** We reviewed serial bioimpedance measurements in order to quantify the relationship between changes in these scores and a patient's risk for developing breast cancer-related lymphedema (BCRL).

***Methods and Results:*** From April 2010 through November 2016, 505 patients were prospectively evaluated using bioimpedance spectroscopy (BIS/L-Dex). Patients received preoperative and postoperative L-Dex measurements and were categorized based upon risk for BCRL with respect to axillary staging procedure, radiation use, taxane use, and body mass index (BMI). L-Dex change was associated with the type and number of BCRL risk factors. Both axillary lymph node dissection (ALND) and regional nodal irradiation (RNI) were associated with a greater change in L-Dex (*p* < 0.001), although elevated BMI was not associated. The median, maximal change in L-Dex for patients treated with ALND, RNI, and taxanes was 16.7 versus 5.2 for ALND alone and 3.7 for sentinel lymph node biopsy (SLNB) alone (*p* = 0.016). In a model using all four risk factors to predict the maximal change in L-Dex, ALND and RNI remained significantly associated with maximum change (*p* < 0.05). The time required to reach maximal change in L-Dex was shorter in patients treated with ALND or RNI (the time for 25% of patients achieving an L-Dex ≥7 was 4.3 months for ALND, RNI, and taxanes patients versus 30.8 months for SLNB-alone patients).

***Conclusions:*** Risk factors for development of BCRL were associated with both the magnitude and timing of change in L-Dex scores. These findings demonstrate the utility of serial L-Dex measurements in providing an objective assessment of a patient's lymphedema status and the value of L-Dex serial measurements to assist in monitoring patients for the development of BCRL. This supports the clinical utilization of serial L-Dex scores to follow patients at risk for BCRL.

## Introduction

Breast cancer is the most common noncutaneous malignancy for women in the United States with over 250,000 new cases diagnosed annually.^1^ Secondary to improved outcomes, the number of breast cancer survivors continues to increase.^2^ As a direct result, a substantial number of women are dealing with the acute, subacute, and chronic toxicities of their treatment, including breast cancer-related lymphedema (BCRL). BCRL can result in either a minor aggravation for some patients or a potentially life-altering side effect that can produce significant morbidity and impairment in quality of life in many others.^3^

The incidence of BCRL is variable and highly dependent on the treatment strategies utilized to manage a patient's stage of disease and can be significantly increased by more aggressive local therapies (mastectomy, axillary dissection, and regional nodal irradiation [RNI]) or by certain systemic therapies.^4–9^ In addition, patient-related factors such as body mass index (BMI) can alter BCRL incidence.^10^ Finally, BCRL incidence rates can be significantly impacted by the diagnostic techniques utilized, with recent studies supporting the use of higher sensitivity diagnostic methods to allow for earlier detection of BCRL. More recently, new data and guidelines (e.g., NCCN) support the application of prospective surveillance programs to detect and monitor BCRL in its subclinical (reversible) phase in order to facilitate early conservative management and prevent progression to its chronic and more costly, irreversible phase.^11–14^

An ideal screening program should employ tools that allow the detection of subclinical BCRL, a phase that cannot be identified with traditional techniques.^3,4,6^ Bioimpedance spectroscopy (BIS) is a relatively newer diagnostic technique that can be used to detect subclinical BCRL in a busy clinic and has limited time, personnel, and space requirements.^11,15–17^ Numerous studies have documented the value of prospective surveillance with BIS and current studies are further clarifying the magnitude of the benefit of its application in reducing BCRL incidence.^18–23^

The underlying premise with the use of BIS is that extracellular fluid changes (as seen with the development of BCRL) predate clinically evident BCRL and can be detected using the technology.^11^ The BIS technique compares the impedance of an at-risk limb to the healthy contralateral one. Early studies documented a conservative normal range for the impedance ratio, which is based upon a healthy population of patients and uses a three-standard deviation (SD) increase from a preoperative/pretreatment baseline to indicate clinical lymphedema (e.g., an L-Dex score ≥10).^11^ Application of the BIS technique and technology has been successfully integrated into many breast cancer survivorship clinics for many years.^18–23^ More recently, the newer concept of subclinical BCRL has been adopted secondary to recent data, documenting reduced rates of chronic BCRL when reversible subclinical lymphedema is detected and conservative early intervention applied.^12,13,24^ The use of this modern strategy employs the application of a lower trigger for intervention using an L-Dex score ≥7.^25^

This analysis was undertaken to help better understand the relationships between the underlying differences in the risk of developing BCRL (based upon a patient's treatment and other factors) and the changes in L-Dex scores seen (both in magnitude and time to development). Theoretically, one would expect that patients who experience the greatest increases in the change in L-Dex scores would be those at greatest risk for developing BCRL. To that end, we present changes in L-Dex scores of 505 women monitored in a large, consistently structured and applied BCRL surveillance protocol versus their underlying risk for developing BCRL (based upon established high-risk factors).

## Materials and Methods

From April 2010 through November 2016, 505 patients were prospectively evaluated with BIS in a structured BCRL surveillance protocol at a single institution (Nashville Breast Center, Nashville, TN) using the L-Dex U400 device (ImpediMed, Brisbane, Australia). Definitive breast surgery (lumpectomy or mastectomy) was required with axillary management, including axillary lymph node dissection (ALND) or sentinel lymph node biopsy (SLNB). Exclusion criteria included bilateral breast cancers, implantable electronic devices (i.e., pacemakers), pregnancy, renal failure, and heart failure.^11^ Data collected included age, BMI, surgical procedure (breast conservation vs. mastectomy), axillary staging (SLNB vs. ALND), endocrine therapy/chemotherapy receipt and type, and use of radiation therapy, including receipt of RNI. Treatment for BCRL was also documented, as was any outcome (clinical and L-Dex scores) following diagnosis. Institutional Review Board (IRB) approval was provided for this retrospective analysis (WIRB Exemption Determination under 45 CFR 46.101(b)(4)).

Patients received a preoperative baseline L-Dex measurement and postoperative measurements at regular intervals (as determined by their physician). The mean, median, and SD change in L-Dex score from pretreatment baseline were calculated for each patient. Patients were categorized into the following BCRL risk groups: (1) all patients, (2) elevated BMI, (3) SLNB, (4) ALND, (5) use of taxanes, (5) use of RNI, (6) those with an elevated BMI, and (7) combinations of SLNB/ALND, RNI, and taxane receipt. Some combinations could not be evaluated due to limited patient numbers. Differences in the magnitude of changes in L-Dex scores based upon high-risk features were calculated and compared, as well as the impact of time on these changes.

### Statistical methods utilized

To assess the association of maximum change in L-Dex from baseline with risk factors, simple linear regression analysis was performed separately for each risk factor. Risk factors were included as binary predictors: ALND versus SLNB, use of taxanes or not, use of RNI versus not, and elevated BMI versus not. To assess the relative strength of each risk factor's association with the maximum change in L-Dex, a multiple linear regression analysis was performed, including all risk factors as predictors in the same model. Kruskal–Wallis test and Wilcoxon rank-sum test were performed to compare the distributions of maximum change in L-Dex among various risk groups. The Kaplan–Meier method was used to estimate the time to achieve 25% of patients reaching L-Dex change of 7 or 10 within each risk group. Statistical analyses were performed using R 3.1.0. R package “survival” was used for Kaplan–Meier estimators. Two-sided *p* values of <0.05 and one-sided *p* values of <0.025 were considered statistically significant.

## Results

### Change in L-Dex versus risk factors for BCRL

The median and mean maximal change with treatment over time in L-Dex from pretreatment baseline was associated with the type and number of risk factors for each patient. For example, ALND (compared with SLNB) and RNI (compared with no RNI) were significantly associated with a greater change in L-Dex individually (*p* < 0.001). The median, maximal change in L-Dex for patients treated with ALND/RNI/taxane was 16.7 versus 5.2 for ALND alone and 3.7 for SLNB (Table 1). The effects of risk factors were additive (i.e., having multiple factors resulted in a greater change than having just one, although not all combinations could be reviewed do to small numbers of patients). In a multiple linear regression model using all four risk factors to predict the maximal change in L-Dex, ALND (compared with SLNB) and RNI (compared with no RNI) still remained significantly associated with greater maximum change during the entire follow-up (*p* < 0.05, Table 2). Risk groups involving ALND and RNI had greater change in L-Dex than any combination of groups without these two factors. Finally, there were significantly higher percentages of patients with changes in L-Dex ≥7 or ≥10 in risk groups involving ALND and RNI (data not shown).

**Table 1.** Median/Mean Change in L-Dex Scores Versus Risk Factors for Breast Cancer-Related Lymphedema

	*All patients (*n* = 505)*	*Elevated BMI (*n* = 4)*	*Taxane (*n* = 100)*	*SLNB only (*n* = 39)*	*SLNB/taxane (*n* = 122)*	*ALND only (*n* = 17)*	*ALND/RNI (*n* = 202)*	*ALND/RNI Taxane (*n* = 7)*
*Median*	3.8	3.2	3.3	3.7	3.6	5.2	9.4	16.7
*Mean*	5.5	4.0	5.4	6.6	5.5	7.4	13.1	17.8

Some combinations of risk factors had too few patients.

ALND, axillary lymph node dissection; BMI, body mass index; RNI, regional nodal irradiation; SLNB, sentinel lymph node biopsy.

**Table 2.** Regression Analysis of Max Change in L-Dex (Entire Follow-Up) on Combined Risk Factors

	*Estimate*	*95%CI lower*	*95%CI upper*	p
ALND	2.32	0.8	3.83	0.02
Taxane	0.2	−0.97	1.36	0.84
RNI	9.76	6.97	12.55	<0.001
Elevated BMI	−0.19	−1.35	0.97	0.85

### Time to maximal change in L-Dex versus risk factors

**FIG. 1.**
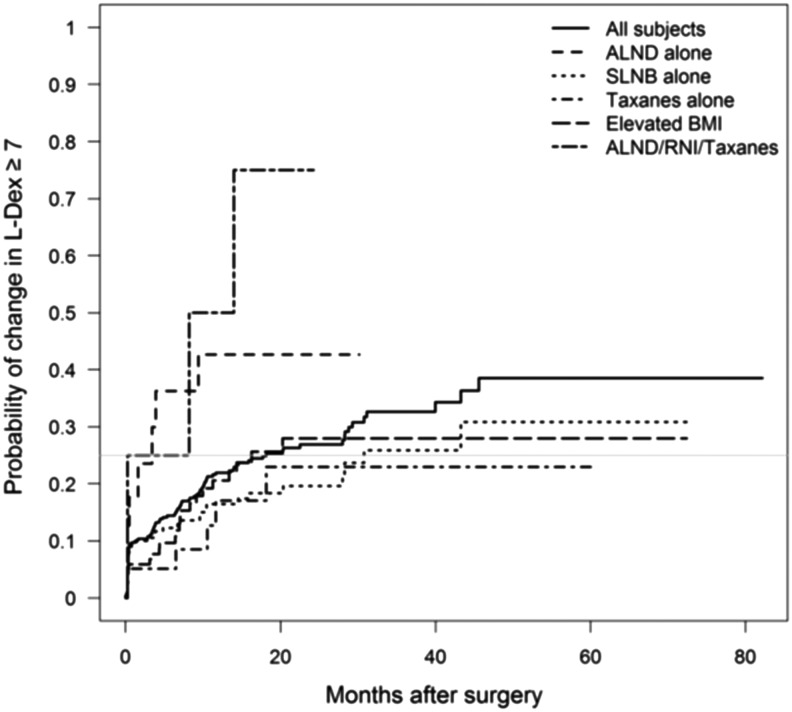
Time to Change in L-Dex Score (>7) based upon risk factors.

**FIG. 2.**
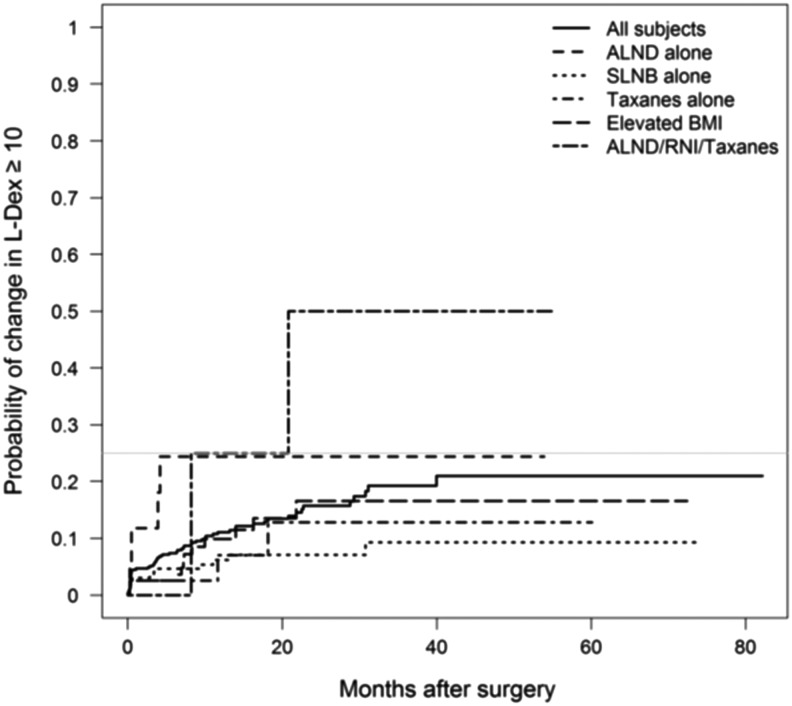
Time to Change in L-Dex Score (>10) based upon risk factors.

The time required to reach the maximal change in L-Dex was substantially shorter in patients treated with ALND or RNI versus patients without either one of these risk factors. For example, the median time for 25% of patients achieving an L-Dex >7 was 4.3 months for ALND/RNI/taxane patients versus 30.8 months for SLNB-alone patients (Fig. 1). The impact of risk factors versus time to failure was also evident when using an L-Dex trigger of ≥10 (Fig. 2).

## Conclusions

This analysis was undertaken to help better understand the relationships of how the underlying differences in the risk of developing BCRL (based upon a patient's treatment) can impact changes in L-Dex scores (both in magnitude and time to development) and how BIS technology tracks extracellular fluid changes potentially leading to BCRL. Physiologically, one would expect that patients who experience the greatest increases in L-Dex scores (impedance values reflecting extracellular fluid accumulation) would be those at greatest risk for developing BCRL. Although patient numbers in some patient risk groups were small because of less frequent contemporary use of aggressive surgical and radiation techniques, the magnitude of change in L-Dex increased as the risk for developing BCRL increased. Patients treated with ALND and RNI had the greatest increase in L-Dex from baseline compared to lower risk patients (SLNB) and the time to reaching these higher scores was substantially less. Conversely, lower risk patients had smaller increases in L-Dex and the time to reach these maximum scores was more prolonged (as demonstrated in Figs. 1 and 2). These observations have important implications for the optimal management of patients and help to better define how to monitor patients based upon their underlying risk for developing BCRL (i.e., frequency of testing). They also help to demonstrate the underlying science of BIS and that L-Dex scores accurately track extracellular fluid changes that can potentially have serious consequences for patients.

### Science of BIS and the use of L-Dex scores in the clinic

The underlying premise with the use of BIS is that extracellular fluid changes (as seen with the development of BCRL) predate clinically evident BCRL and can be detected using this technology.^11^ The BIS technique compares the impedance of an at-risk limb to the healthy contralateral limb. As discussed previously, earlier studies established a conservative normal range for the impedance ratio from a healthy population and used a three SD increase from a preoperative baseline as an indicator of clinical lymphedema (e.g., L-Dex score >10).^17^ Application of the technique and technology has been successfully integrated and used in the detection and management of BCRL for many years.^18–23^ Unfortunately, until this analysis, very few publications utilizing BIS in a large, structured surveillance program have been available to confirm the accuracy of the technology (L-Dex scores) to track the anticipated risk of BCRL. Well-accepted high-risk factors for BCRL were shown to clearly be correlated with BIS, confirming the basic science of BIS as it relates to extracellular fluid accumulation and its impact on BCRL.

### Defining a lower threshold for intervention using L-Dex scores

As noted above, when BIS was initially developed for use as a lymphedema measurement tool, the concept of subclinical lymphedema was not yet described in the scientific literature. More recently, this concept of subclinical BCRL has been promoted because of data showing reduced rates of chronic BCRL if reversible subclinical lymphedema is detected and early intervention applied^12,13,24,26,27^ (Table 3). Contemporary use of this strategy now employs the application of a lower trigger for intervention using an L-Dex score ≥7.

**Table 3.** Studies Evaluating Early Detection and Intervention for Breast Cancer-Related Lymphedema

	*Type of study*	*Year published*	*Patients*	*BCRL diagnostic technique/intervention*	*Results*
Box et al.^27^	Randomized	2002	65	Circumference, bioimpedance/early physiotherapy	Early intervention reduced BCRL (30% vs. 11%)
Torres Lacomba et al.^13^	Randomized	2010	120	Circumference/early physiotherapy	Early intervention reduced BCRL (25% vs. 7%)
Stout et al.^12^	Prospective	2011	196	Perometry/compression garment	46 patients developed BCRL; 25% subclinical lymphedema, 6% advanced BCRL (Stage I/II)
Soran et al.^26^	Prospective	2014	186	Bioimpedance/physical therapy, compresson garment, education	33% subclinical BCRL, early intervention reduced clinical BCRL (36% vs. 4%)

BCRL, breast cancer-related lymphedema.

Based upon the work of Stout et al., the concept of subclinical lymphedema and early intervention was initially demonstrated.^12^ This novel study suggested that early intervention with a simple compression sleeve might prevent chronic, irreversible lymphedema if it is applied in the subclinical range. Stout et al. advocated that a >3% volume change postoperatively was indicative of subclinical lymphedema; also, BIS, was recommended as a possible diagnostic method to evaluate and detect subclinical lymphedema.^12^ Shortly after the publication of the Stout study, BIS was indeed put forth as a desirable measurement method to identify subclinical lymphedema.^5^ This concept was based primarily on work performed by Ward et al., which found BIS to be more sensitive than tape measurement in detecting BCRL.^6^

Recent publications now advocate that subclinical lymphedema is actually present when there is a change in the L-Dex reading of ≥7, representative of two SDs above the norm established in early studies.^15,25,26,28^ Fu et al. found that an L-Dex ratio of ≥7.1 helped delineate between breast cancer patients with BCRL and those at risk, with ∼20% of patients with lymphedema missed with a 7.1 cutoff.^25^ Taken together these data support the need to diagnose subclinical BCRL with an L-Dex score less than seven to allow for early intervention.^18–23^ Consistent with this, the currently accruing phase III trial, evaluating BIS compared to tape measurements, has adopted a lower threshold to initiate early intervention.^29^ Finally, it is important to note that the association of L-Dex scores with risk factors for BCRL noted in this analysis was consistent, regardless of the trigger chosen to diagnose subclinical lymphedema. However, differences in the time to detect BCRL were obviously significantly impacted depending upon the trigger chosen (Figs. 1 and 2).

### Study limitations

A major limitation of this analysis is the lack of sufficient numbers of patients in some high-risk categories, making it difficult to accurately quantify the magnitude of each of these factors on the change in L-Dex scores. This obviously reflects the trends in the contemporary management of breast cancer patients, where less aggressive surgical and radiation techniques are employed. Nonetheless, the overall impact of their use (i.e., ALND and RNI) still remains evident on L-Dex changes accurately reflecting the countless publications documenting their potential detrimental effect on the incidence of BCRL.
